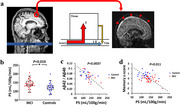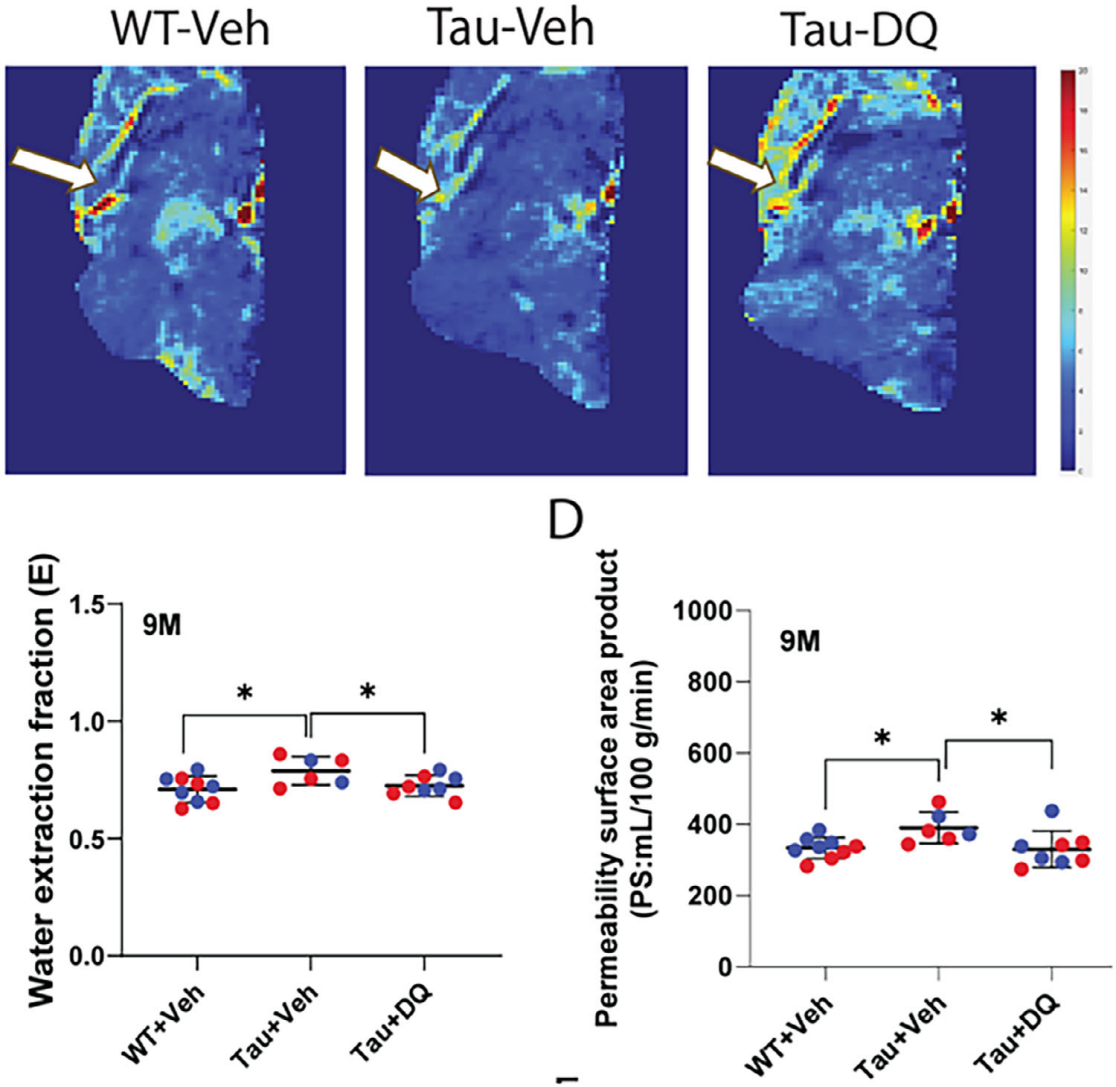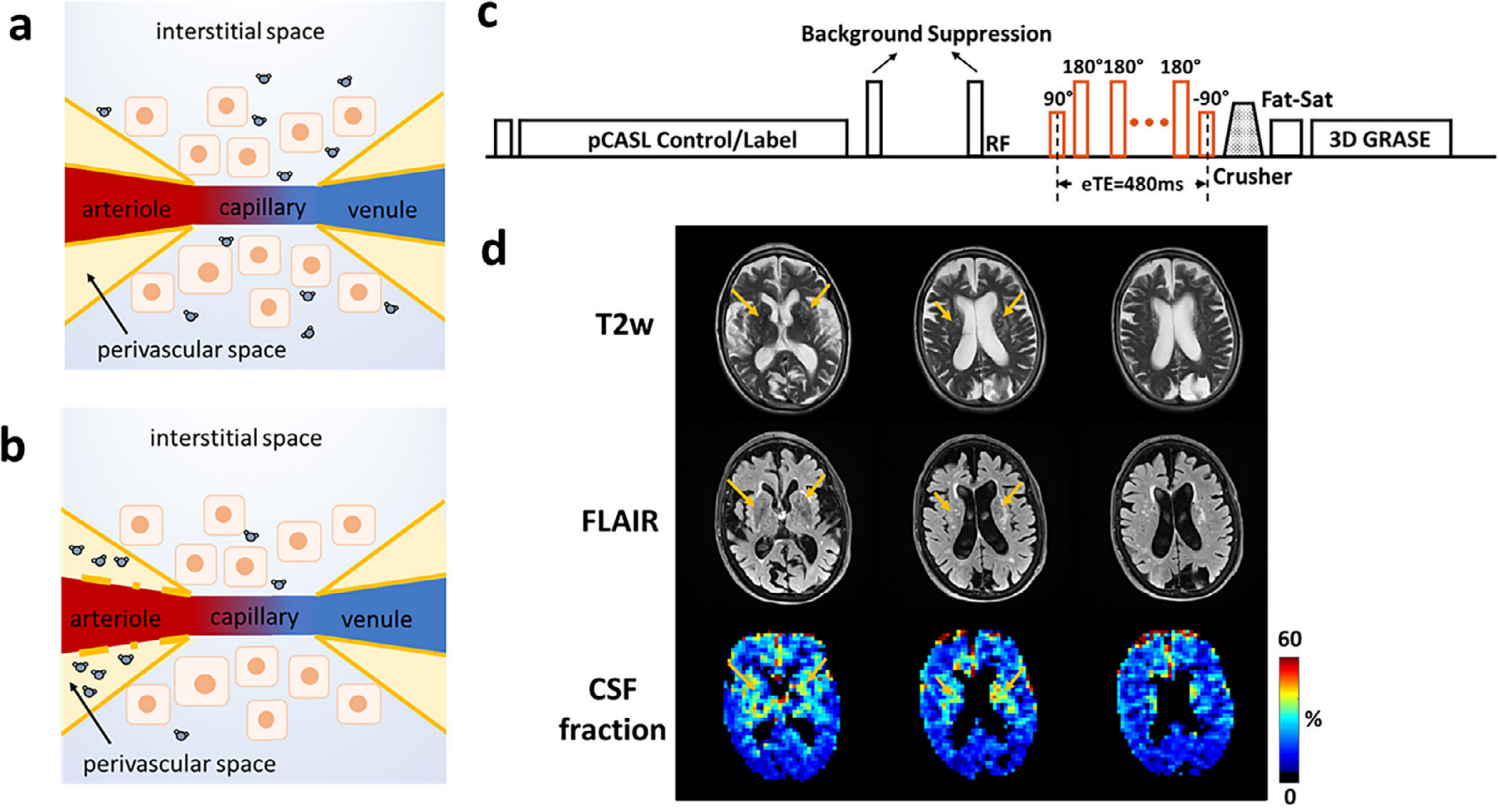# Non‐contrast MRI of blood‐brain barrier, arterial vessel integrity, glymphatic function in ADRD and ARIA

**DOI:** 10.1002/alz70856_102611

**Published:** 2025-12-25

**Authors:** Hanzhang Lu

**Affiliations:** ^1^ Johns Hopkins University School of Medicine, Baltimore, MD, USA

## Abstract

**Background:**

Neurofluid exchange in the brain is critical for its normal function. Abnormalities in these exchanges are associated various clinical manifestations in the Alzheimer's disease and related dementia (ADRD) spectrum. This presentation will discuss recent advances in MRI imaging technologies to assess neurofluid exchanges at multiple interfaces and their applications in ADRD including amyloid related imaging abnormalities (ARIA).

**Method:**

We will discuss three aspects of neurofluid exchange, measured with non‐contrast MRI. Section 1 will describe imaging of blood‐brain barrier (BBB) permeability using non‐contrast MRI. MRI can measure BBB's permeability to water at the capillary‐tissue interface. Section 2 will discuss imaging of arterial vessel integrity using MRI, which can potentially serve as a surrogate marker for microbleed risks and ARIA. Section 3 will discuss the assessment of water exchange between interstitial fluid (ISF) and perivascular space (PVS) fluid, potentially related to glymphatic function.

**Result:**

BBB permeability to water was found to be elevated in patients with mild cognitive impairment (MCI), the degree of which was associated with CSF measures of beta‐amyloid 42 and ptau (Figure 1). BBB permeability was found to be related to cognitive function, in particular memory domain scores. Studies in animal models of AD confirmed these findings. Mouse models of 5xFAD and tauopathy revealed increased BBB permeability, which was confirmed by biotin tracer and could be reverted by senolytic treatment (Figure 2). ARIA is primarily attributed to arterial vessel wall breakdown, leading to proteins (ARIA‐E) or red blood cells (ARIA‐H) leaking out of vessels. Arterial‐spin‐labeling (ASL) MRI under long echo‐time (TE) can selectively measure water molecules leaking out of arteries and entering perivascular space (Figure 3). Thus, hyperintensities in long‐TE ASL images may be indicative of ARIA risks (Figure 3). On the other hand, diminished signals (hypointensities) in long‐TE ASL images may indicate reduced ISF and PVS exchange and abnormal glymphatic function. Normative long‐TE ASL images in younger and older healthy subjects have been established.

**Conclusion:**

Recent advances in MRI technologies allowed the assessment of neurofluid exchange at several interfaces. These techniques can provide important insights on the role of neurofluid in the pathogenesis of neurodegenerative diseases.